# Characteristic of entire corneal topography and tomography for the detection of sub-clinical keratoconus with Zernike polynomials using Pentacam

**DOI:** 10.1038/s41598-017-16568-y

**Published:** 2017-11-28

**Authors:** Zhe Xu, Weibo Li, Jun Jiang, Xiran Zhuang, Wei Chen, Mei Peng, Jianhua Wang, Fan Lu, Meixiao Shen, Yuanyuan Wang

**Affiliations:** 1 School of Ophthalmology and Optometry, Wenzhou Medical University, Wenzhou, Zhejiang China; 2 Department of Ophthalmology, Bascom Palmer Eye Institute, University of Miami, Miami, Florida USA; 3 Key Laboratory of Adaptive Optics, Chinese Academy of Sciences, Chengdu, Sichuan China; 4 Institute of Optics and Electronics, Chinese Academy of Sciences, Chengdu, Sichuan China

## Abstract

The study aimed to characterize the entire corneal topography and tomography for the detection of sub-clinical keratoconus (KC) with a Zernike application method. Normal subjects (n = 147; 147 eyes), sub-clinical KC patients (n = 77; 77 eyes), and KC patients (n = 139; 139 eyes) were imaged with the Pentacam HR system. The entire corneal data of pachymetry and elevation of both the anterior and posterior surfaces were exported from the Pentacam HR software. Zernike polynomials fitting was used to quantify the 3D distribution of the corneal thickness and surface elevation. The root mean square (RMS) values for each order and the total high-order irregularity were calculated. Multimeric discriminant functions combined with individual indices were built using linear step discriminant analysis. Receiver operating characteristic curves determined the diagnostic accuracy (area under the curve, AUC). The 3rd-order RMS of the posterior surface (AUC: 0.928) obtained the highest discriminating capability in sub-clinical KC eyes. The multimeric function, which consisted of the Zernike fitting indices of corneal posterior elevation, showed the highest discriminant ability (AUC: 0.951). Indices generated from the elevation of posterior surface and thickness measurements over the entire cornea using the Zernike method based on the Pentacam HR system were able to identify very early KC.

## Introduction

Iatrogenic keratectasia is the most feared complication after corneal refractive surgery^1,2^. With the exception of surgery-caused factors, such as excess ablation of corneal tissues and thin residual stromal beds, corneas with undetected keratoconus (KC) or pellucid marginal degeneration (PMD) are known to be at high risk for iatrogenic keratectasia^3–5^. Thus, detection of KC in its earliest stage is very important for the preoperative screening of corneal refractive surgery. KC is a bilateral corneal ectasia disease that is characterized by a progressive thinning of the cornea, with an increase in the anterior and posterior corneal curvature^6^. Advanced KC can be easily diagnosed using corneal topography and a slit lamp. However, it is still a challenge to precisely distinguish a preclinical KC cornea from a normal cornea before refractive surgery, due to the lack of early-stage symptoms^7^. Those with suspected bilateral KC often do not present typical signs and symptoms until a definitive KC develops in one eye^8^. This may be the main reason for the difficulty in diagnosing sub-clinical KC in current clinical practices. Some evidence show that the asymptomatic fellow eye of unilateral KC, termed as subclinical, or forme fruste KC, had great potential to progressing clinical KC^7–11^. Therefore, evaluating and characterizing the topography and tomography features of the corneas in these particular eyes may help clinicians to improve screening methods in order to distinguish suspected KC cases from normal corneas and prevent iatrogenic keratectasia after refractive surgery.

Placido disc-based corneal topography can detect localized steeping in the anterior cornea surface the topography, which is considered to be the first detectable clinical sign of KC^12^. However, many studies have demonstrated that only the anterior topographic changes of the cornea may not be a strong enough indicator of early KC^11,13^. In contrast to the Placido disc-based corneal topography, Scheimpflug-based topography allows assessment of the anterior and posterior corneal surface shapes and corneal tomography^14^. Based on the information on corneal topography and tomography provided by Scheimpflug-based tomography technology, several methods have been introduced to quantitatively identify the early KC from normal eyes^9,15^. According to previous studies, the regular mathematical formulations were limited to quadratic or cubic-order ploynomials, which may reduce the corneal 3D distribution by averaging the results as a function of both radius and meridian^14,16^. The Zernike coefficients based on Zernike fitting on the corneal surface configuration and thickness can be used to characterize the 3D map varying complexity of corneal shapes and spatial distribution of corneal thickness^17^, which may provide a highly sensitive and specific diagnostic tool for the early detection of KC^14,17–19^.

The goal of the present study was to apply the Zernike fitting method to describe the 3D varying complexity of corneal shapes and the 3D distribution of corneal thickness, and to characterize the entire corneal topography and tomography data in sub-clinical eyes, KC eyes, and normal eyes using Pentacam tomography. Furthermore, the metrics constructed from Zernike polynomials were compared to improve the diagnostic sensitivity and specificity for the detection of sub-clinical KC corneas.

## Results

### Demographics

Fifty eyes of 50 normal subjects (28 men and 22 women, average age 24.5 ± 2.3 years), 28 eyes of 28 sub-clinical KC patients (22 men and 6 women, average age 21.8 ± 5.5 years), and 49 eyes of 49 KC patients (29 men and 20 women, average age 24.6 ± 6.0 years) were enrolled to build discrimination functions . The validation set of patients included 97 eyes of 97 normal subjects (56 men and 41 women, average age 24.1 ± 2.6 years), 49 eyes of 49 sub-clinical KC patients (40 men and 9 women, average age 22.8 ± 5.7 years), 90 eyes of 90 KC patients (73 men and 17 women, average age 23.8 ± 5.4 years). Table 1 display the demographic information, the KISA% value and correlated ingredient values. The values of the I-S value and the SRAX value showed significant difference between normal group and sub-clinical KC group (one way analysis of variance [ANOVA] least significant difference [LSD] test, P < 0.05, Table 1). As to the parameters with significant difference for KC and the normal group from the Pentacam HR system, the astigmatism keratometry and average keratometry in the KC group were significantly higher than the other two groups (one way ANOVA LSD test, P < 0.05, Table 1). However, the results of the astigmatism keratometry and average keratometry showed no statistical differences in sub-clinical eyes compared with normal group. The thinnest corneal thickness (TCT), the maximum anterior elevation (AEmax) and the maximum posterior elevation (PEmax) from the Pentacam HR system showed significant difference between normal group and sub-clinical KC group (one way ANOVA LSD test, P < 0.05, Table 1).

**Figure 1:**
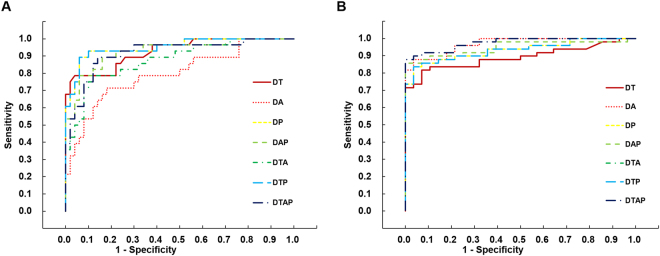
Receiver operating characteristic (ROC) curves of discriminant functions for normal, sub-clinical KC, and KC groups. (**A**) ROC curve of the output value of discriminant functions for sub-clinical KC group versus normal group. (**B**) ROC curve of the output value of discriminant functions for KC group versus sub-clinical KC group.

**Table 1:** Clinical information of all subjects for normal, sub-clinical KC, and KC groups.

Group (eyes/subjects)	Normal (147/147)	Sub-clinical KC (77/77)	KC (139/139)
**Demographics**			
Spherical equivalent (D)	−3.93 ± 2.26	−3.68 ± 3.25	−6.85 ± 4.14^†*^
(95%CI)	(−4.29 to −3.56)	(−4.41 to −2.95)	(−7.54 to −6.16)
Astigmatism keratometry (D)	1.26 ± 0.67	1.46 ± 0.70	5.14 ± 3.13^†*^
(95%CI)	(1.15 to 1.37)	(1.30 to 1.62)	(4.61 to 5.66)
Average keratometry (D)	43.63 ± 1.41	43.51 ± 1.13	48.72 ± 4.42^†*^
(95%CI)	(43.40 to 43.86)	(43.26 to 43.77)	(47.98 to 49.46)
BCVA (decimal VA)	1.06 ± 0.10	1.01 ± 0.10	0.56 ± 0.27^†*^
(95%CI)	(1.04 to 1.07)	(0.99 to 1.04)	(0.51 to 0.60)
**Medmont system**			
K	43.72 ± 1.60	43.78 ± 1.26	49.53 ± 4.86^†*^
(95%CI)	(43.46 to 43.98)	(43.49 to 44.06)	(48.72 to 50.34)
I-S	−0.11 ± 0.62	0.28 ± 0.71*	3.32 ± 2.00^†*^
(95%CI)	(−0.21 to −0.01)	(0.12 to 0.44)	(2.99 to 3.66)
AST	1.33 ± 0.72	1.49 ± 0.68	4.52 ± 2.23^†*^
(95%CI)	(1.22 to 1.45)	(1.34 to 1.65)	(4.15 to 4.90)
SRAX	24.10 ± 28.84	46.70 ± 50.87*	94.77 ± 49.12^†*^
(95%CI)	(19.43 to 28.78)	(35.26 to 58.14)	(86.58 to 102.97)
KISA%	9.25 ± 12.40	22.87 ± 34.48*	2192.87 ± 3857.85^†*^
(95%CI)	(7.24 to 11.26)	(15.12 to 30.62)	(1549.20 to 2836.54)
**Pentacam HR system**			
Anterior flatkeratometry (D)	42.82 ± 1.29	42.57 ± 1.19	49.45 ± 18.53^†*^
(95%CI)	(42.61 to 43.03)	(42.30 to 42.84)	(46.36 to 52.55)
Anterior steep keratometry (D)	43.91 ± 1.58	43.96 ± 1.27	52.18 ± 6.60^†*^
(95%CI)	(43.65 to 44.16)	(43.67 to 44.25)	(51.08 to 53.29)
Anterior average keratometry (D)	43.35 ± 1.38	43.25 ± 1.15	50.00 ± 5.98^†*^
(95%CI)	(43.13 to 43.58)	(42.99 to 43.51)	(49.00 to 50.99)
Anterior maximum keratometry (D)	44.71 ± 1.65	45.11 ± 1.45	58.60 ± 9.26^†*^
(95%CI)	(44.44 to 44.98)	(44.78 to 45.43)	(57.06 to 60.15)
Posterior flat keratometry (D)	6.08 ± 0.40	6.12 ± 0.29	7.16 ± 1.04^†*^
(95%CI)	(6.02 to 6.15)	(6.06 to 6.18)	(6.98 to 7.33)
Posterior steep keratometry (D)	6.45 ± 0.26	6.48 ± 0.29	8.02 ± 1.25^†*^
(95%CI)	(6.40 to 6.49)	(6.42 to 6.55)	(7.81 to 8.23)
Posterior average keratometry (D)	6.27 ± 0.22	6.29 ± 0.27	7.56 ± 1.12^†*^
(95%CI)	(6.24 to 6.31)	(6.23 to 6.35)	(7.37 to 7.75)
TCT	530.42 ± 25.44	508.11 ± 32.39*	446.92 ± 49.81^†^*
(95%CI)	(526.29 to 534.55)	(500.83 to 515.40)	(438.61 to 455.23)
AEmax	3.80 ± 1.59	6.37 ± 2.69*	29.05 ± 15.80^†^*
(95%CI)	(3.54 to 4.06)	(5.76 to 6.98)	(26.41 to 31.69)
PEmax	8.00 ± 2.82	16.12 ± 7.59*	63.83 ± 33.49^†*^
(95%CI)	(7.55 to 8.46)	(14.41 to 17.83)	(58.24 to 69.41)

Normal, normal group; Sub-clinical KC, sub-clinical keratoconus group; KC, keratoconus group; BCVA, best corrected visual acuity; 95% CI, 95% confidence interval.

^*^Compared to the normal group P < 0.05 by one way ANOVA LSD test.

^†^Compared to the sub-clinical KC group P < 0.05 by one way ANOVA LSD test.

### Intergroup Differences: Zernike Polynomials Fitting for Corneal Elevation of Anterior and Posterior Surfaces and Corneal Pachymetry

Compared with the normal group, there were significant differences in the 3rd, 5th, 6th, and higher-order irregularity coefficients (HOI) root mean square (RMS) values of the anterior corneal surface elevation derived from Zernike polynomials analysis in the sub-clinical KC group (one way ANOVA LSD test, P < 0.05, Table 2). All the RMSs of anterior surface discriminated between sub-clinical KC and normal eyes with the values of the area under (AUC) the receiver operating characteristic (ROC) curve were higher than 0.638 and lower than 0.751 (Table 2).

**Table 2:** The diagnostic indices with significant difference for sub-clinical KC and KC.

Diagnostic indices	Normal	Sub-clinical KC					KC				
	Mean	Mean	AUC	cut off	Sen (%)	Spe (%)	Mean	AUC	cut off	Sen (%)	Spe (%)
**Anterior surface**											
3rd order RMS	1.587 ± 0.729	2.631 ± 1.440^*^	0.751	1.852	68	78	11.473 ± 5.825^†*^	0.974	4.992	88	93
5th order RMS	0.428 ± 0.260	0.602 ± 0.237^*^	0.749	0.455	71	70	2.262 ± 1.219^†*^	0.978	1.084	88	100
6th order RMS	0.284 ± 0.154	0.403 ± 0.171^*^	0.734	0.351	61	86	1.255 ± 1.225^†*^	0.917	0.577	84	86
HOI RMS	3.268 ± 0.584	3.816 ± 1.239^*^	0.638	3.720	39	90	13.413 ± 7.088^†*^	0.980	6.302	90	96
**Corneal pachymetry**											
3rd order RMS	2.439 ± 0.716	5.774 ± 2.301^*^	0.926	3.829	79	96	14.697 ± 7.351^†*^	0.891	8.596	82	94
5th order RMS	1.302 ± 0.388	1.836 ± 1.023^*^	0.698	1.436	68	72	3.913 ± 1.923^†*^	0.805	2.805	74	93
HOI RMS	4.489 ± 0.776	7.142 ± 2.723^*^	0.824	5.587	68	92	18.266 ± 8.842^†*^	0.904	11.757	80	96
**Posterior surface**											
3rd order RMS	3.299 ± 1.232	8.248 ± 3.631^*^	0.928	4.597	86	88	27.362 ± 13.153^†*^	0.947	13.474	90	93
5th order RMS	1.569 ± 0.499	2.318 ± 1.291^*^	0.704	1.704	64	68	6.404 ± 3.233^†*^	0.952	3.896	80	96
HOI RMS	8.314 ± 1.214	11.037 ± 3.715^*^	0.699	10.156	54	96	32.646 ± 16.324^†*^	0.911	14.991	92	90
**Medmont system**											
I-S	−0.072 ± 0.689	0.287 ± 0.673^*^	0.681	0.245	64	71	3.254 ± 1.988†^*^	0.996	1.905	95	100
SRAX	24.265 ± 27.270	49.357 ± 54.621^*^	0.612	8.500	85	35	89.500 ± 51.090^†*^	0.737	32.500	85	64
KISA%	10.373 ± 14.210	19.492 ± 23.139^*^	0.645	9.267	54	74	2144.832 ± 3883.942^†*^	0.959	97.000	81	100
**Pentacam HR system**											
TCT	530.873 ± 25.567	509.036 ± 29.902^*^	0.695	498.835	92	47	450.667 ± 41.148^†*^	0.862	479.5	83	78
AEmax	3.896 ± 1.636	6.155 ± 2.507^*^	0.812	4.585	72	73	27.466 ± 14.182^†*^	0.974	11.165	92	93
PEmax	7.953 ± 2.989	16.167 ± 7.185^*^	0.856	12.335	93	67	60.748 ± 29.935^†*^	0.970	30.165	94	88

Normal, normal group; Sub-clinical KC, sub-clinical keratoconus group; KC, keratoconus group; AUC, area under receiver operating characteristic curve; Sen, sensitivity; Spe, specificity; RMS, the values of root mean square; HOI, higher-order irregularity.

^*^Compared to the normal group P < 0.05 by one way ANOVA LSD test.

^†^Compared to the sub-clinical KC group P < 0.05 by one way ANOVA LSD test.

As to the Zernike fitting of the entire corneal pachymetry distribution, the 3rd, 5th, and HOI RMSs showed significant differences between the sub-clinical KC group and the control group (one way ANOVA LSD test, P < 0.05, Table 2). All the AUCs of RMSs were higher than 0.698 and lower than 0.926 in sub-clinical KC discrimination (Table 2).

The Zernike polynomials modeling metrics for the cornea elevation of the posterior surface was statistically significant between the sub-clinical KC and the control group in the 3rd, 5th, and HOI RMSs (one way ANOVA LSD test, P < 0.05, Table 2). Among these individual metrics, the 3rd-order RMS of the posterior elevation obtained the highest discriminating capability (AUC: 0.928; sensitivity = 86%; specificity = 88%) in sub-clinical KC eyes (Table 2).

### Discriminant Analysis

The formulas for all discriminant functions are included in Table 3. The discriminant functions (D) consist of the Zernike RMSs and HOIs of corneal thickness (T), anterior surface (A), and/or posterior surface (P). The output values of the discriminant functions were significantly different between the three groups (one way ANOVA LSD test, P < 0.05, Table 4, Figure 1). With comparison of the single metrics obtained from Zernike polynomials modeling, the discriminant functions improved the diagnostic power of each anterior surface (DA, AUC = 0.798, Table 4, Figure 1), posterior surface (DP, AUC = 0.951, Table 4, Figure 1), and corneal thickness (DT, AUC = 0.927, Table 4, Figure 1), respectively. However, the combination of the anterior surface with the posterior surface (DAP, AUC = 0.926, Table 4, Figure 1), corneal thickness (DTA, AUC = 0.876, Table 4, Figure 1) and all metrics (DTAP, AUC = 0.918, Table 4, Figure 1) did not improve the diagnostic power. The function DP, which was derived from the posterior surface Zernike coefficients of sub-clinical KC and normal eyes and consisted of the RMS values of the 3rd and total higher-order coefficients, had the highest discriminant ability (AUC = 0.951, sensitivity: 89%, specificity: 94%).

**Table 3:** Formulas of the discriminant functions.

Discriminant Functions	Formulas
DT	0.212^*^ T_3rd order_ − 1.675
DA	0.862^*^ A_3rd order_ + 1.04^*^ A_6th order_ − 0.64^*^ A_HOI_ − 0.887
DP	−0.077^*^ P_HOI_ + 0.203^*^ P_3rd order_ − 1.37
DTA	0.675^*^ A_3rd order_ + 0.770^*^ A_6th order_ − 0.518^*^ A_HOI_ + 0.086^*^ T_3rd order_ − 1.229
DTP	−0.077^*^ P_HOI_ + 0.203^*^ P_3rd order_ − 1.37
DAP	0.116^*^ A_3rd order_ + 0.989^*^ A_6th order_ − 0.251^*^ P_HOI_ + 0.307^*^ P_3rd order_ − 0.942
DTAP	0.148^*^ T_HOI_ + 0.257^*^ A_3rd order_ + 0.816^*^ A_6th order_ − 0.307^*^ P_HOI_ + 0.238^*^ P_3rd order_ − 1.189

A, the anterior surface; P, the posterior surface; T, the corneal thickness; certain order, the root mean square of certain order; HOI, the root mean square of total higher-order irregularity.

**Table 4:** The output values of discrimination functions constructed from Zernike RMS metrics with significant differences for sub-clinical KC and KC.

Discrimination	Normal	Sub-clinical KC					KC				
functions	mean	mean	AUC	cut off	Sen (%)	Spe (%)	mean	AUC	cut off	Sen (%)	Spe (%)
DT	−1.158 ± 0.152	−0.451 ± 0.488^*^	0.927	−0.863	79	96	1.441 ± 1.558^†*^	0.891	0.148	83	93
DA	−1.316 ± 0.464	−0.641 ± 0.626^*^	0.798	−0.931	71	82	1.723 ± 1.469^†*^	0.967	0.180	87	96
DP	−1.340 ± 0.209	−0.546 ± 0.475^*^	0.951	−1.040	89	94	1.671 ± 1.549^†*^	0.933	0.191	85	96
DTA	−1.381 ± 0.414	−0.540 ± 0.808*	0.876	−1.021	79	88	1.787 ± 1.509^†*^	0.970	0.262	91	96
DTP	−1.340 ± 0.209	−0.546 ± 0.475^*^	0.951	−1.040	89	94	1.671 ± 1.549^†*^	0.933	0.191	85	96
DAP	−1.551 ± 0.419	−0.476 ± 0.598^*^	0.926	−1.117	89	84	1.835 ± 1.478^†*^	0.946	0.574	87	100
DTAP	−1.653 ± 0.461	−0.552 ± 0.627^*^	0.918	−1.179	89	86	1.976 ± 1.458^†*^	0.974	0.530	87	100

Normal, normal group; Sub-clinical keratoconus, sub-clinical keratoconus group; KC, keratoconus group; AUC, area under receiver operating characteristic curve; Sen, sensitivity; Spe, specificity.

^*^Compared to the normal group P < 0.05 by one way ANOVA LSD test.

^†^Compared to the sub-clinical KC group P < 0.05 by one way ANOVA LSD test.

### Validation results

The accuracy and precision results of the discrimination function were improved with the data generated from the posterior corneal surface and corneal thickness due to the Zernike fitting. The value of true accuracy was always more than 82.2% for the sub-clinical KC discrimination (Table 5). The true prediction was more than 73.2% for the sub-clinical KC discrimination (Table 5). The sensitivity and specificity were higher when both corneal surfaces and pachymetry metrics were included for the sub-clinical KC diagnosis. The DTAP formula showed higher sensitivity (83.7%) and specificity (84.5%). In contract, the individual anterior surface metrics obtained from corneal topography, such as I-S, SRAX and KISA%, showed a reduction for the sub-clinical KC discrimination (Table 5).

**Table 5:** Confusion matrix (actual vs predicted group), accuracy, precision, sensitivity, specificity of the diagnostic indices and discrimination functions performed on the validation set.

	Actual Group	Total	Predicted Group		Accuracy (%)	Precision (%)	Sensitivity (%)	Specificity (%)
			Normal	Sub-clinical KC				
**DT**	**Normal**	97	84	13	82.2	73.5	73.5	86.6
	**Sub-clinical KC**	49	13	36				
**DA**	**Normal**	97	82	15	79.5	69.4	69.4	84.5
	**Sub-clinical KC**	49	15	34				
**DP**	**Normal**	97	86	11	84.2	77.1	75.5	88.7
	**Sub-clinical KC**	49	12	37				
**DTA**	**Normal**	97	86	11	84.9	77.6	77.6	88.7
	**Sub-clinical KC**	49	11	38				
**DTP**	**Normal**	97	86	11	84.2	77.1	75.5	88.7
	**Sub-clinical KC**	49	12	37				
**DAP**	**Normal**	97	78	19	80.8	67.8	81.6	80.4
	**Sub-clinical KC**	49	9	40				
**DTAP**	**Normal**	97	82	15	84.2	73.2	83.7	84.5
	**Sub-clinical KC**	49	8	41				
**I-S**	**Normal**	97	76	21	71.9	58.0	59.2	78.4
	**Sub-clinical KC**	49	20	29				
**SRAX**	**Normal**	97	34	63	51.4	39.4	83.7	35.1
	**Sub-clinical KC**	49	8	41				
**KISA%**	**Normal**	97	75	22	69.9	55.1	55.1	77.3
	**Sub-clinical KC**	49	22	27				
**TCT**	**Normal**	97	89	8	77.4	75.0	49.0	91.8
	**Sub-clinical KC**	49	25	24				
**AEmax**	**Normal**	97	71	26	72.6	57.4	71.4	73.2
	**Sub-clinical KC**	49	14	35				
**PEmax**	**Normal**	97	90	7	83.6	82.1	65.3	92.8
	**Sub-clinical KC**	49	17	32				

Normal, normal group; Sub-clinical KC, sub-clinical keratoconus group; KC, keratoconus group.

## Discussion

In order to detect the early or mild signs of KC, many investigators have tried to define specific and objective indices to quantitatively describe the topographic and tomographic characteristics of sub-clinical KC corneas^20–25^. This is particularly important for ruling out early KC when screening candidates for refractive surgery to reduce the risk of ectasia and prevent IK. However, it is still an ongoing challenge to identify the earliest KC stages with absolute accuracy. Recently, a global consensus was reached among the ophthalmology experts from four supranational corneal societies. The consensus standardized the definition of corneal ectasia procession, which included at least two of the following parameters: steepening of the anterior corneal surface, steepening of the posterior corneal surface, and progressive thinning and/or an increase in the rate of corneal thickness change from the periphery to the thinnest point^25^. Therefore, studying the characteristics of both the corneal topography and tomography can be of valuable interest. To our knowledge, this is the first study to comprehensively describe the properties of the entire 3D corneal topography and tomography in sub-clinical KC eyes using the Zernike polynomials modeling method. For the anterior and posterior corneal surfaces, the Zernike polynomials were used to fit the anterior and posterior elevation data to define the varying complexity of corneal shapes^18,19^. Similarly, Zernike polynomials modeling was used to analyze the thickness data in order to describe the 3D thickness distribution with sub-micrometer accuracy^14^. Previous works have proven that the indices based on this method were significantly different in KC corneas compared to normal corneas^14,17–19^. Our results further demonstrated that varying complexity of corneal shapes in the mildest stage of KC disease (sub-clinical KC), especially for the posterior surface, is higher compared to normal eyes. The 3D corneal thickness distribution was also significantly different between normal eyes and sub-clinical KC eyes. These results suggest that Zernike polynomials fitting for the tomography data from Pentacam HR system provides useful information for discriminating the early stage of KC disease.

There was no significant difference in the flat keratometry, steep keratometry, average keratometry, and maximum keratometry of anterior and posterior surfaces from the Pentacan HR system between normal eyes and sub-clinical eyes (Table 1), although those indices were significantly higher in KC groups. Similar to several previous studies, our results indicate that these single indices based on axial curvature were not sensitive enough to study the earliest abnormal shape in the mildest stage of KC^9,15^. There were several ways to express the early abnormal shape of KC cornea based on elevation data from the corneal surfaces obtained by Pentacam rotating Scheimpflug camera^9,15,26,27^. The Zernike polynomials application to the corneal surfaces shows the varying complexity of corneal shapes^17,28^. Some investigators have reported that the indices based on this method were significantly different in KC corneas than in normal corneas^18,19^. Interestingly, in our study, the 3rd, 5th, and HOI RMS of the anterior and posterior surfaces using Zernike polynomials fitting were significantly higher in both sub-clinical KC and KC groups than that in normal eyes. The overall predictive accuracy of these indices of both the anterior and posterior surfaces was high for KC with AUC values > 0.90. However, this level of varying complexity in corneal shapes for the anterior surface was not sufficient to exceed the threshold for positive sub-clinical KC with the normal corneas (AUC < 0.80). For the posterior surface, the 3rd RMS value reached a high accuracy in separating the subclinical KC cornea from the normal cornea (AUC > 0.90). However, the indices of the posterior surface were also less effective in discriminating sub-clinical KC than they were in discriminating KC. On the other hand, the discriminating power of 3rd order RMS of corneal pachymetry was higher between the sub-clinical KC and normal eyes than between the sub-clinical KC and KC. This may indicate that the pachymetry varies more earlier during the KC progressing, which could be extended to evaluate effect of early KC managements, such as cross-linking and rigid gas permeable (RGP) lenses^14^.

It was suggested that progressive thinning of the cornea may be another sign of the earliest stage of KC. Therefore, several indices were calculated based on corneal thickness to express the localized thinning, such as the difference of focal minimum thinning and maximum thickness (Min-Max), the difference of the inferotemporal (IT) octant and superonasal (SN) octant (IS-IT), and the cone location magnitude index (CLMI)^11,29–31^. However, these mathematical indices reduced the 3D distribution of corneal thickness spatial variation, which formulated in both the radius and themeridian^14,16^. Recently, with an improvement in the technique, corneal thickness distribution has been leveraged to define new indices to assess the rate of thickness change in the cornea from the thinnest point (cone center) to the periphery to detect KC and sub-clinical KC^11,22,32^. Although such indices expressed the trend in thickness variation, the quantitative results were not sufficient to capture the corneal complexity changes. The method of Zernike polynomials fitting was a newly developed method to describe the entire 3D corneal thickness distribution with a high degree of spatial resolution^14^. Shetty *et al*. showed that the indices based on this method had good accuracy in detecting KC cornea^14^. We also found a significantly larger value for indices of the 3rd, 5th, and HOI for both KC and sub-clinical KC eyes. The predictive accuracy of these indices was high for KC (AUC > 0.90). Our study further indicates that the Zernike polynomials method based on the entire 3D corneal thickness was able to capture the mildest pachymetric changes with high resolution.

In general, based on data from the Pentacam HR system, the Zernike polynomials modeling of anterior surface elevation data did not discriminate the sub-clinical KC eyes from normal eyes as well as posterior surface data, although individual index and discrimination function DA accurately separated the KC eyes from sub-clinical eyes. The AUC values of DT and DP showed higher diagnostic power between the sub-clinical KC and normal eyes, which were agreed with results of TCT and PEmax. Similar results were reported in previous studies, which referred that the indices of posterior elevation and pachymetric changes were useful for discriminating early KC^14,26^. However, there was no single anterior surface RMS value which classified the sub-clinical KC and normal eyes with sensitively ≥80%. In addition, the output values of the discrimination function DA did not separate as well as the output values from the posterior surface data functions DP (Table 4). The AUC, sensitivity, and specificity did not improve, or even dropped minimally, if anterior surface data were included in the discrimination function. This result strongly suggests that the anterior surface elevation data obtained alone from the Pentacam HR system is not suitable for the diagnosis of sub-clinical KC. This notion has also been discussed in previous studies^27^. Using the same Pentacam HR tomography system, Bae *et al*. reported that the anterior elevation difference was less sensitive and specified for the sub-clinical KC discrimination compared with the posterior elevation difference^27^. Reddy *et al*. used the dual Scheimpflug analyzer (Ziemer USA, Wood River, IL) and also found that the posterior elevation was more useful than the anterior elevation for the sub-clinical KC diagnosis^33^. However, Jafarinasab *et al*. used Orbscan II topography (Bausch and Lomb, Rochester, NY, USA) found that the anterior elevation was more workable for sub-clinical KC determination^34^. Based on the Orbscan topography results, Buhren *et al*. used the similar Zernike polynomials application. They concluded that the Zernike metrics of the anterior corneal surface were more sensitive for the sub-clinical KC differentiation^18,19^. This might be due to the different technical principles used in the Orbscan and Pentacam systems. The Orbscan system is a slit-scanning based corneal topography, which was reported to have lower repeatability and reproducibility in measuring corneal thickness and posterior elevation compared with the Scheimpflug-based tomography^35,36^ Additionally, due to a slightly shorter capturing time and an imaging registration method using central corneal points, the Pentacam device showed higher repeatability and reproducibility compared to the Orbscansystem^32^. The surface results of different corneal topography and tomography were not interchangeable. Above all, more attention should be paid when different commercial systems of corneal topography and tomography are used for sub-clinical KC diagnosis in daily clinical practice.This study showed that indices generated from entire corneal thickness and surfaces were able to identify very mild forms of KC, which could not detected by Placido topography. According to previous study, the KISA% value was able to filter the suspect KC and KC from normal cornea. The KISA% index was highly likely to identify the KC stage with the value over 100%. As to the suspect KC, the KISA% was proved to be 60%^12^. The current study also showed that the cut-off value of KISA% was 97.0 for the KC diagnosis, which was similar with the previous study. However, the KISA% was not efficient to discrimination the sub-clinical KC cornea. This might due the sub-clinical KC was more tend to be normal corneal with I-S less than 1.4, compared with the suspect KC with I-S more than 1.4 and less than 1.9^7^. This result also agreed that any single or combined indices may be insufficient to identify a suspect cornea from a normal one, as the reported indices showed some degree of overlap in normal and sub-clinical corneas. In order to verify the diagnostic accuracy of the studied indices for suspect cornea identification, a longitudinal study with a larger sample size is necessary. In addition, further study is needed to verify whether our approach could detect other corneal conditions that tend to develop into keratectasia.

Despite the new findings in detecting early KC eyes using topographic and tomographic tools, biomechanics changes caused by corneal ectasia are assumed the predisposing factors^37,38^. Ocular response analyzer (ORA) (Reichert Ophthalmic Instruments Inc, Buffalo, NY, USA), which can report the indices such as the corneal hysteresis (CH) and the corneal resistance factor (CRF), are useful for the measurements of corneal biomechanics and strength^37,38^. Several studies have determined that CH and CRF were significantly lower in KC eyes than in normal eyes. However, the CH and CRF were not powerful indices for discriminating mild KC from normal eyes. The role of central corneal thickness might be the limited factor which is not yet clearly defined^39,40^. By using the ultra-high-speed Scheimpflug camera, the Corvis ST (Oculus Optikgeräte GmbH, Wetzlar, Germany) system can capture the corneal dynamic changes and biomechanical properties *in vivo*. Vinciguerra *et al*. built the Corvis Biomechanical Index (CBI) included dynamic corneal response parameters using the Corvis ST system. The CBI allowed high sensitivity and specificity for distinguishing KC from normal eyes^41^. Ambrósio *et al*. combined the Scheimpflug based corneal tomography and the Corvis ST system together for enhancing corneal ectasia detection^42^. The integrated Tomographic and Biomechanical Index (TBI) they built provided greater accuracy for detecting mild corneal ectasia^42^. Mercer *et al*. developed the Dynamic Corneal Response (DCR) index included biomechanical properties parameters collected by the Corvis ST system. The DCR index was also able to separate healthy from keratoconic eyes^43^. The indices combined the corneal tomography with dynamic biomechanical response improved the diagnostic ability for sub-clinical KC and KC eyes^44,45^. In the future studies, the Zernike polynomials application in the current study combined with the corneal biomechanics detection might be further validated for the early KC discrimination.

In summary, indices generated from 3D thickness and elevation of posterior surface measurements over the entire cornea using Zernike polynomials fitting based on Pentacam HR system enabled the detection of sub-clinical KC, which may not be detected by Placido-based topography. The Zernike method may be a useful tool to capture the subtle changes of topography and tomography at the very early stages of KC. In future, a longitudinal study with a larger sample size needs to be conducted for further validation of the Zernike polynomials application in the detection of corneas at risk of developing keratectacisa.

## Methods

### Study Population

The Office of Research Ethics, Wenzhou Medical University, approved the study. Written informed consent was obtained from all patients after the purpose and characteristics of the study were well explained. The tenets of the Declaration of Helsinki were followed for all the research procedures. KC patients were enrolled at the Affiliated Eye Hospital of Wenzhou Medical University in China. Complete ocular examinations were performed by experienced doctors (JJ and WC), including a review of medical and family history, corrected distance visual acuity, slit-lamp biomicroscopy, fundus examination, and corneal topography using Medmont E300 (Medmont, Inc., Nunawading Melbourne, Australia). The original topography data were outputted and tabulated in an Excel spread sheet (Microsoft, Redmond, USA). A custom-developed MATLAB^®^-based (MathWorks, Inc., Natick, MA, USA) software was used to quantified the KISA%, which was derived from 4 indices: the K-value, an expression of central corneal steepening; the I-S value, an expression of vertical asymmetry; the AST value, an expression of corneal regular astigmatism; the SRAX value, an expression of corneal irregular astigmatism^12^.

The subjects were assigned to three groups. KC Group (mild or moderate KC eyes): (1) central average keratometry > 47.0D;(2) at least one of the following slit-lamp signs: stromal thinning, Vogt’s strias, Fleischer’s ring > 2-mm arc; (3) asymmetric topographical features with I-S ≥1.9 diopter (D) of the vertical gradient power across the 6-mm region; and (4) no history of contact lens wear, ocular surgery, or extensive scarring. Sub-clinical KC Group (fellow eye of unilateral KC): (1) central average keratometry <45.0D; (2) a diagnosis of KC in the contralateral eye; (3) no clinical signs of KC at slit-lamp biomicroscopy, retinoscopy, and ophthalmoscopy; (4) corneal topographical features with I-S values < 1.4D of the vertical gradient power across the 6-mm region; and (5) no history of contact lens wear, ocular surgery, or trauma. Normal Group (healthy eyes from normal subjects) were enrolled from the hospital staff and university students if they met the following screening criteria: (1) central average keratometry <45.0D; (2) myopia <−6.00 D and astigmatism <−2.00 D; (3) no clinical signs or suggestive topographic patterns for suspicious sub-clinical KC, KC, or pellucid marginal degeneration; (4) no history of ocular surgery or trauma; and (5) stopped contact lens wear for ≥8 weeks for rigid gas permeable and ≥4 week for soft contact lenses. All the patients were divide into a training set (normal, sub-clinical KC group and KC group) used to build the discrimination function, and a validation set (normal and sub-clinical group) used to test the diagnostic power.

### Study Procedure

Corneal tomographic examinations were performed with a Pentacam HR system (Oculus, GmbH, Wetzlar, Germany). The subjects were required to place their chin on the chin rest and their forehead against the forehead strap. The operator held the joystick and adjusted it following the direction on screen. Each subject was asked to blink once completely to spread an optically smooth tear film on the cornea before scanning. Patients were instructed to keep both eyes open while the blue light scanned for about 2 seconds. Only when “Examination Quality Specification” showed “OK” were the corneal pachymetry results accepted. Three repeated measurements were obtained from each subject. The built-in Pentacam HR software (version 6.02r23) was used to export the raw data of the entire corneal elevation and pachymetric distribution. The AEmax, the PEmax over an 8-mm best-fit sphere and TCT were read from the software interface. The U12 files containing the raw data of anterior interface elevation, posterior interface elevation and corneal pachymetry mappings from Pentacam HR system were exported. The data were transferred and tabulated in an Excel spread sheet using custom-developed MATLAB^®^-based software.

### Zernike Polynomials Fitting Analysis

The Zernike polynomials method was performed to fit the maps of the anterior and posterior corneal surface elevations and corneal thickness measured by Scheimpflug imaging^46^. Different from using the reference bodies to stimulate human corneal surface and pachymetry, the Zernike polynomials was directly fitted on the raw data of the 3D corneal elevation and pachymetry^13,47^. The Zernike terms outputted from the Zernike polynomials, such as Zernike coefficients and the root mean square, were used for further analysis^14,48^. The analysis zone was set as a 6-mm diameter around the corneal vertex. The Zernike coefficients up to 7th-order were obtained and the root mean square (RMS) of each order and total higher-order irregularity coefficients (HOI) were calculated. The Zernike polynomials was expressed as:

1 $$W(\rho ,\,\theta )=\sum _{{\rm{i}}=0}^{n}{C}_{m}^{i}{Z}_{m}^{i}$$

where *W* is the wavefront error, *ρ* is the nondimensional radius (0 ≤ *ρ* ≤ 1), *θ* is the meridian in radians, *C* is the Zernike coefficient, *Z* is the Zernike polynomial, *i* is the order of the Zernike polynomial, *m* is from −*n*, *−n+2*, …*to*…, *n* − *2*, *n*.

The RMS is defined as the square root of the mean of the squared differences between the local measured (*W*), and the mean estimated ($\hat{W}$) at all the data points on the elevation and pachymetry maps were computed:

2 $${W}_{RMS}=\sqrt{\frac{{\sum }_{j-1}^{k}{({W}_{j}-{\hat{W}}_{j})}^{2}}{k}}$$

where *W*_*RMS*_ is the root mean square of the Zernike coefficients, *j* is the ordering number of the Zernike polynomials, and *k* is the count of the Zernike coefficients.

### Statistical Analysis

ALL of the data analyses were performed using the Statistical Package for the Social Sciences software (ver. 17, SPSS, Inc., Chicago, IL, USA). Based on the 3 repeated acquirements using Pentacam HR system, each measurement was performed with Zernike polynomials fitting analysis. The Zernike polynomials fitting result of single subject was averaged by the 3 repeated analyses. All the data were analyzed by the one-way analysis of variance (ANONA). The significance of difference between the groups (normal, sub-clinical KC, and KC groups) was subjected to the least significant difference (LSD) test. P < 0.05 was considered a statistically significant difference.

In order to build discriminant functions with the lowest possible number of individual RMSs of each order, HOIs, and Pentacam metrics, linear stepwise discriminant analysis was applied to build discriminant functions. Metrics screened by the Mahalanobis distance from the centroid normal group were included in the function. The discriminant functions were constructed from Zernike RMS metrics for the anterior and posterior elevations and corneal pachymetry with statistical significances between the sub-clinical KC group and the normal groupas follows:

DT = Zernike RMSs and HOIs of corneal thickness

DA = Zernike RMSs and HOIs of anterior surface

DP = Zernike RMSs and HOIs of posterior surface

DAP = Zernike RMSs and HOIs of anterior and posterior surface

DTA = Zernike RMSs and HOIs of anterior surface and corneal thickness

DTP = Zernike RMSs and HOIs of posterior surface and corneal thickness

DTAP = Zernike RMSs and HOIs of anterior and posterior surfaces and corneal thickness

The output values of the discriminant functions were evaluated to differentiate between the control and subclinical KC groups, and the control and KC groups.

The ROC curves were used to value the diagnostic power of each individual index and the output values of the discriminant function in differentiating between KC and normal corneas and between sub-clinical KC and normal corneas. Sensitivity and specificity were calculated for the optimum cutoff values obtained from ROC curves. An area under the ROC curve (AUC) of 100% implied perfect diagnostic performance^49^.

## References

[1] Haw WW, Manche EE (2001). Iatrogenic keratectasia after a deep primary keratotomy during laser *in situ* keratomileusis. Am. J. Ophthalmol..

[2] Tervo TM (2001). Iatrogenic keratectasia after laser *in situ* keratomileusis. J. Cataract Refract. Surg..

[3] Randleman JB, Russell B, Ward MA, Thompson KP, Stulting RD (2003). Risk factors and prognosis for corneal ectasia after LASIK. Ophthalmology.

[4] Ambrósio R, Wilson SE (2002). Early pellucid marginal corneal degeneration: case reports of two refractive surgery candidates. Cornea.

[5] Amoils SP, Deist MB, Gous P, Amoils PM (2000). Iatrogenic keratectasia after laser *in situ* keratomileusis for less than −4.0 to −7.0 diopters of myopia. J. Cataract Refract. Surg..

[6] Rabinowitz YS (1998). Keratoconus. Surv. Ophthalmol..

[7] Klyce SD (2009). Chasing the suspect: keratoconus. Br. J. Ophthalmol..

[8] Li X, Rabinowitz YS, Rasheed K, Yang H (2004). Longitudinal study of the normal eyes in unilateral keratoconus patients. Ophthalmology.

[9] Labiris G (2014). Diagnostic capacity of the keratoconus match index and keratoconus match probability in subclinical keratoconus. J. Cataract Refract. Surg..

[10] De SU (2008). Sensitivity and specificity of posterior corneal elevation measured by Pentacam in discriminating keratoconus/subclinical keratoconus. Ophthalmology.

[11] Steinberg J (2015). Screening for Subclinical Keratoconus Using Swept-Source Fourier Domain Anterior Segment Optical Coherence Tomography. Cornea.

[12] Rabinowitz YS, Rasheed K (1999). KISA% index: a quantitative videokeratography algorithm embodying minimal topographic criteria for diagnosing keratoconus. J. Cataract Refract. Surg..

[13] De Sanctis U, Aragno V, Dalmasso P, Brusasco L, Grignolo F (2013). Diagnosis of subclinical keratoconus using posterior elevation measured with 2 different methods. Cornea.

[14] Shetty R (2015). A novel zernike application to differentiate between three-dimensional corneal thickness of normal corneas and corneas with keratoconus. Am. J. Ophthalmol..

[15] Muftuoglu O, Ayar O, Hurmeric V, Orucoglu F, Kilic I (2015). Comparison of multimetric D index with keratometric, pachymetric, and posterior elevation parameters in diagnosing subclinical keratoconus in fellow eyes of asymmetric keratoconus patients. J. Cataract Refract. Surg..

[16] Ambrósio R (2011). Novel pachymetric parameters based on corneal tomography for diagnosing keratoconus. J. Refract. Surg..

[17] Smolek MK, Klyce SD (2005). Goodness-of-prediction of Zernike polynomial fitting to corneal surfaces. J. Cataract Refract. Surg..

[18] Buhren J, Kuhne C, Kohnen T (2007). Defining subclinical keratoconus using corneal first-surface higher-order aberrations. Am. J. Ophthalmol..

[19] Buhren J, Kook D, Yoon G, Kohnen T (2010). Detection of subclinical keratoconus by using corneal anterior and posterior surface aberrations and thickness spatial profiles. Invest Ophthalmol. Vis. Sci..

[20] Rabinowitz YS, Rasheed K (1999). KISA% index: a quantitative videokeratography algorithm embodying minimal topographic criteria for diagnosing keratoconus. J. Cataract Refract. Surg..

[21] Buhren J, Kuhne C, Kohnen T (2007). Defining subclinical keratoconus using corneal first-surface higher-order aberrations. Am. J. Ophthalmol..

[22] Belin MW, Khachikian SS (2007). Corneal diagnosis and evaluation with the OCULUS Pentacam. Highlights of Ophthalmology.

[23] Ambrósio R, Alonso RS, Luz A, Coca Velarde LG (2006). Corneal-thickness spatial profile and corneal-volume distribution: tomographic indices to detect keratoconus. J. Cataract Refract. Surg..

[24] Ambrósio R, Klyce SD, Wilson SE (2003). Corneal topographic and pachymetric screening of keratorefractive patients. J. Refract. Surg..

[25] Gomes JA (2015). Global consensus on keratoconus and ectatic diseases. Cornea.

[26] De Sanctis U (2008). Sensitivity and specificity of posterior corneal elevation measured by Pentacam in discriminating keratoconus/subclinical keratoconus. Ophthalmology.

[27] Bae GH (2014). Corneal topographic and tomographic analysis of fellow eyes in unilateral keratoconus patients using Pentacam. Am. J. Ophthalmol..

[28] Carvalho LA (2005). Accuracy of Zernike polynomials in characterizing optical aberrations and the corneal surface of the eye. Invest Ophthalmol. Vis. Sci..

[29] Mahmoud AM (2013). Expanding the cone location and magnitude index to include corneal thickness and posterior surface information for the detection of keratoconus. Am. J. Ophthalmol..

[30] Rabinowitz YS, Li X, Canedo AL, Ambrosio R, Bykhovskaya Y (2014). Optical coherence tomography combined with videokeratography to differentiate mild keratoconus subtypes. J. Refract. Surg..

[31] Li Y (2008). Keratoconus diagnosis with optical coherence tomography pachymetry mapping. Ophthalmology.

[32] Tajbakhsh Z (2012). Comparison of keratometry measurements using the Pentacam HR, the Orbscan IIz, and the TMS-4 topographer. Ophthalmic Physiol Opt..

[33] Reddy JC (2014). Comparative evaluation of dual Scheimpflug imaging parameters in keratoconus, early keratoconus, and normal eyes. J. Cataract Refract. Surg..

[34] Jafarinasab MR (2015). Sensitivity and specificity of posterior and anterior corneal elevation measured by orbscan in diagnosis of clinical and subclinical keratoconus. J. Ophthalmic Vis. Res..

[35] Quisling S, Sjoberg S, Zimmerman B, Goins K, Sutphin J (2006). Comparison of Pentacam and Orbscan IIz on posterior curvature topography measurements in keratoconus eyes. Ophthalmology.

[36] Crawford AZ, Patel DV, McGhee CN (2013). Comparison and repeatability of keratometric and corneal power measurements obtained by Orbscan II, Pentacam, and Galilei corneal tomography systems. Am. J. Ophthalmol..

[37] Schweitzer C (2010). Screening of forme fruste keratoconus with the ocular response analyzer. Invest Ophthalmol. Vis. Sci..

[38] Goebels S (2015). Staging of keratoconus indices regarding tomography, topography, and biomechanical measurements. Am. J. Ophthalmol..

[39] Mohammadpour M (2015). Ocular response analyzer parameters in healthy, keratoconus suspect and manifest keratoconus eyes. Oman. J. Ophthalmol..

[40] Fontes BM, Ambrosio R, Jardim D, Velarde GC, Nose W (2010). Corneal biomechanical metrics and anterior segment parameters in mild keratoconus. Ophthalmology.

[41] Vinciguerra R (2016). Detection of Keratoconus With a New Biomechanical Index. J. Refract. Surg..

[42] Ambrósio R (2017). Integration of Scheimpflug-Based Corneal Tomography and Biomechanical Assessments for Enhancing Ectasia Detection. J. Refract. Surg..

[43] Mercer RN (2017). Comparison of Corneal Deformation Parameters in Keratoconic and Normal Eyes Using a Non-contact Tonometer With a Dynamic Ultra-High-Speed Scheimpflug Camera. J. Refract. Surg..

[44] Wang YM, Chan TCY, Yu M, Jhanji V (2017). Comparison of Corneal Dynamic and Tomographic Analysis in Normal, Forme Fruste Keratoconic, and Keratoconic Eyes. J. Refract. Surg..

[45] Elham R (2017). Keratoconus diagnosis using Corvis ST measured biomechanical parameters. J. Curr. Ophthalmol..

[46] Thibos LN, Applegate RA, Schwiegerling JT, Webb R (2002). Standards for reporting the optical aberrations of eyes. J. Refract. Surg..

[47] Sideroudi H, Labiris G, Giarmoukakis A, Bougatsou N, Kozobolis V (2014). Contribution of reference bodies in diagnosis of keratoconus. Optom. Vis. Sci..

[48] Iskander DR, Collins MJ, Davis B (2001). Optimal modeling of corneal surfaces with Zernike polynomials. IEEE Trans. Biomed. Eng.

[49] Altman DG, Bland JM (1994). Diagnostic tests 3: receiver operating characteristic plots. BMJ.

